# Obstructive Shock, from Diagnosis to Treatment

**DOI:** 10.31083/j.rcm2307248

**Published:** 2022-06-29

**Authors:** Viviane Zotzmann, Felix A. Rottmann, Katharina Müller-Pelzer, Christoph Bode, Tobias Wengenmayer, Dawid L. Staudacher

**Affiliations:** ^1^Interdisciplinary Medical Intensive Care (IMIT), Medical Center - University of Freiburg, Faculty of Medicine, University of Freiburg, 79106 Freiburg, Germany; ^2^Department of Medicine IV, Faculty of Medicine, University of Freiburg, 79106 Freiburg, Germany; ^3^Department of Diagnostic and Interventional Radiology, Faculty of Medicine, University of Freiburg, 79106 Freiburg, Germany; ^4^Department of Cardiology and Angiology I, Heart Center Freiburg University, Faculty of Medicine, University of Freiburg, 79106 Freiburg, Germany

**Keywords:** obstructive shock, circulatory shock, review

## Abstract

Shock is a life threatening pathological condition characterized by inadequate tissue oxygen supply. Four different subgroups of shock have been proposed according to the mechanism causing the shock. Of these, obstructive shock is characterized by reduction in cardiac output due to noncardiac diseases. The most recognized causes include pulmonary embolism, tension pneumothorax, pericardial tamponade and aortic dissection. Since obstructive shock typically cannot be stabilized unless cause for shock is resolved, diagnosis of the underlying disease is eminent. In this review, we therefore focus on diagnosis of obstructive shock and suggest a structured approach in three steps including clinical examination, ultrasound examination using the rapid ultrasound in shock (RUSH) protocol and radiological imaging if needed.

## 1. Introduction

### 1.1 Definition of Obstructive Shock

In general, shock is a circulatory failure that results in inadequate cellular oxygen utilization [1]. According to the mechanism causing the shock, four different types have been defined, including cardiogenic, hypovolemic, distributive and obstructive shock based on a classification by Cox and Hinshaw [2] from 1972. The classification of shock has hardly changed over the past few decades [3, 4]. Common to all types of shock is a mismatch of oxygen supply and consumption, which ultimately leads to poor perfusion and multiple organ failure [5]. Three components affect cardiac output: the blood volume, the cardiac output and vascular resistance [6]. A significant disturbance in any of these three factors may result in a critical undersupply of the whole body, a state which is defined as shock [1]. While cardiogenic and obstructive shock both result in shock due to undersupply with blood [7], it is important to distinguish between those two entities since cardiogenic shock is caused by primary cardiac dysfunction [7] while in obstructive shock is caused by extra cardiac diseases (like cardiac tamponade). The etiology of shock is of immense importance since treatment and underlying diseases differ. The most frequent causes of obstructive shock are given in Table 1 and include pulmonary embolism, pneumothorax and cardiac tamponade.

**Table 1.** **Causes of obstructive shock**.

Cause	Effect	Pathology	Pathology
		Intravasal/intraluminal	Extravasal/extraluminal
Disorders of diastolic filling	RV-Preload ↓		∙ Tension pneumothorax
			∙ Cardiac tamponade
			∙ Caval compression syndrome
			∙ Ventilation with high PEEP
Obstruction in the pulmonary circulation	RV-Afterload ↑	∙ Pulmonary embolism	∙ Pulmonary compression syndrome by mediastinal mass
	LV-Preload ↓	∙ Intracardiac mass	
Obstruction in the aortic circulation	LV-Afterload ↑	∙ Leriche Syndrome	∙ Aortic dissection

Abbreviations: RV, right ventricle; LV, left ventricle; PEEP, positive end-expiratory pressure.

### 1.2 Epidemiology

There are no reliable data on the frequency of obstructive shock. The incidences of the most common causes of obstructive shock are given below and can serve as a surrogate for obstructive shock incidence. A population-based incidence for aortic dissection suggests an incidence of 2.3–16.3/100,000 inhabitants per year [8]. Venous thromboembolism, which includes thrombosis of deep leg and pelvic veins and pulmonary embolism is much more frequent and varies between 100–200/100,000 inhabitants per year. Of these, about one third present with pulmonary embolism [9]. The overall person consulting rate for pneumothorax (primary and secondary combined) in the Great Britain was 24.0/100,000 per year for men and 9.8/100,000 for women [10]. A pericardial effusion is a frequent finding in patients with pulmonary hypertension, AIDS or malignoma. In a study of patients presenting in the emergency department with unexplained dyspnea, 13.6% had a pericardial effusion [11]. However, the frequency of obstructive shock cannot be derived from the incidence of these underlying diseases alone. Therefore, more data is required on this matter in order to understand the incidence of obstructive shock.

### 1.3 Pathophysiology

**Fig. 1.**
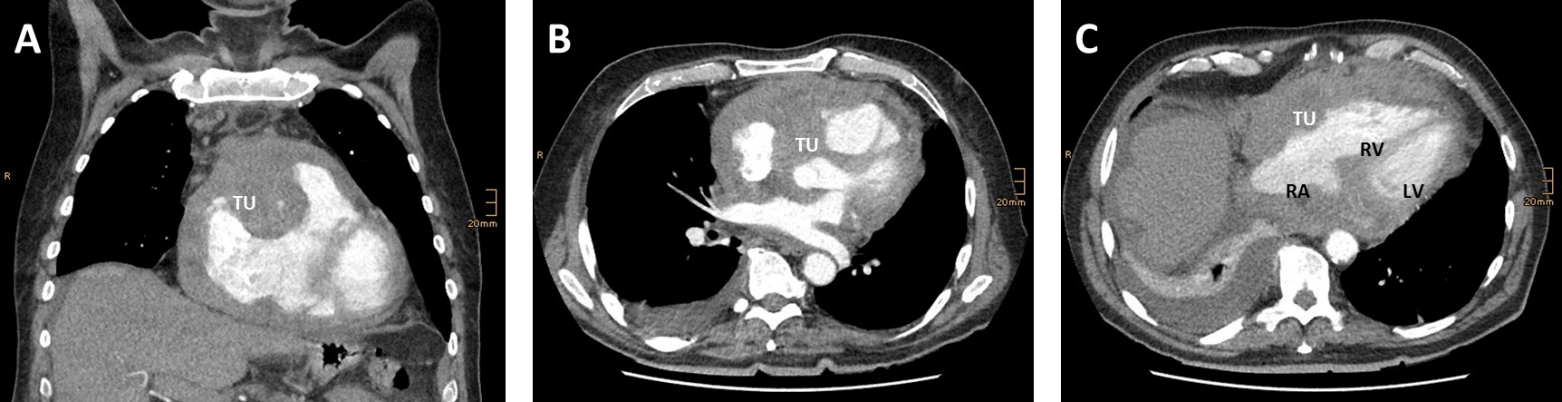
**Transversal (A,B) and coronal (C) reconstructions of a computed tomography (CT) angiography shwoeing a rare cause for obstructive shock**. (A) Tumor mass (TU) infiltrating the right ventricular wall and left ventricular ouitflow tract. (B) The tumor (TU) mass compresses the right artrium (RA) and right ventricle (RV). The tumor has no direct contact to the left ventricle (LV). (C) Demonstrates tumor grophs along the right heart in coronal reconstruction. Adapted from [12].

Etiologically, obstructive shock is caused by an impaired diastolic filling and thus a reduced cardiac RV- or LV-preload (venous return). A reduced preload is caused by tension pneumothorax, V. cava compression syndrome, mediastinal tumors, pericardial effusion or ventilation with a very high PEEP level. On the other hand, diseases which lead to an increased afterload and thus cardiac output may lead to obstructive shock. Causes associated with an increase in afterload are, for example, an aortic dissection, pulmonary embolism or Leriche syndrome. A pulmonary embolism or mediastinal space-occupying mass increases right-ventricular afterload, while decreasing left ventricular preload. The same mechanisms may occur in case of obstructive intracardial mass as displayed in Fig. 1, (Ref. [12]). Obstruction of the aortic blood flow however increases left ventricular afterload (e.g., Leriche syndrome [aortoiliac occlusive disease], aortic dissection) [13].

## 2. Prognoses

The prognosis of obstructive shock strictly depends on the appropriate treatment of the underlying cause and the extent of the tissue damage which had occurred by the time the shock was treated. Since no resilient data on obstructive shock mortality is available, mortality rates of the three most common causes of obstructive shock are discussed below.

### 2.1 Tension Pneumothorax

Prognosis depends on the extend of the tension component (valve mechanism) and how quickly therapy is initiated. Without treatment, a fulminant tension pneumothorax is fatal due to a cardiac arrest. If a tension pneumothorax is treated appropriately however, prognosis of the pneumothorax itself is very good. Based on the cause-of-death statistics, disease-specific mortality for spontaneous pneumothorax is 0.094/100,000 population annually, with a lethality of 0.7% [14], and might be even higher in secondary pneumothorax. Mortality rates in Great Britain between 1950–1997 were 1.26/million per year for men and 0.62/million per year for women [10]. It is important however to consider that most of these patients were not in obstructive shock.

### 2.2 Pulmonary Embolism

According to an epidemiological study from 6 European countries, more than 317,000 deaths were associated with pulmonary embolism in 2004 [15]. Of these, 34% presented with fatal pulmonary embolism; 59% of the deaths were caused by pulmonary embolism that had not been diagnosed during lifetime. The average mortality rate from pulmonary embolism is given as 11% (40,000 deaths/year in Germany), but is significantly higher in patients in shock (40–50%) or in patients requiring resuscitation (70–80%) [15].

### 2.3 Aortic Dissection 

Due to the high preclinical mortality and missing forensic and clinical autopsy data, the number of unreported cases can safely be assumed to be very high [8]. 26% of the patients who receive surgical treatment due to a type A dissection die within the clinic. The mortality of the conservatively treated patients was reported 58% [9].

## 3. Diagnosis

### 3.1 Anamnesis 

The diagnostic algorithm in case of shock is guided by patient history [16] and is a crucial part of the diagnostic workup. Typical patient histories for pulmonary embolism include previous arterial embolisms, thrombosis, malignant disease, immobilization or surgery in the preceding four weeks [17]. Cardiac tamponade is suspected primarily in trauma patients as possible sequel of rupture of the heart [18] and those who have a history of previous, slowly accumulating cardiac effusion related to preconditions such as viral infections, tuberculosis, uremia or neoplasia [19] or in patients after heart surgery [20]. Tension pneumothorax (and hemothorax) must be suspected especially in trauma patients [21] while spontaneous pneumothorax rarely causes relevant intrathoracic tension [22]. In aortic dissection, sudden onset of chest, back or abdominal pain is the most common symptom and often combined with a neurological deficit as a consequence of reduced cerebral perfusion [9]. Ventilation with high PEEP should also be considered as possible reason for vascular obstruction [23]. An obstructive shock by mediastinal mass can only be suspected in patients with known intrathoracic tumor such as lymphomas. Collapse of large vessels or airways by a mediastinal mass is a known problem under general anesthesia and should be prepared for accordingly [24].

### 3.2 Clinical Examination

#### 3.2.1 Physical Examination

Clinical findings in shock include shivering, paleness of skin, arterial hypotension, tachycardia, centralization, dyspnea, hypoxemia, impaired mental status including syncope and reduced urine output and can all rapidly lead up to circulatory death [16, 23, 25]. Thus clinical findings specific for different types of obstructive shock are crucial to direct rapid treatment [23]. If an upper inflow congestion (bulging neck veins, cyanotic-livid complexion) is present, obstructive shock should be suspected (pneumothorax, cardiac tamponade). Clinical examination can also guide towards Leriche syndrome (lack of foot, popliteal and femoral pulses, cold legs on both sides, pale lower extremities). A juxtaposition of focal neurological symptoms and shock can hint towards aortic dissection.

#### 3.2.2 Auscultation 

Decreased or missing respiratory sounds on one hemithorax are typical signs of pneumo- and hemothorax [26]. In auscultation of the heart Pulsus paradoxus as a possible correlate of obstructive shock was described as early as 1873 in the context of constrictive pericarditis [27]. Pulsus paradoxus describes a pathological increase in the physiological decrease of systolic blood pressure during inspiration and includes the extreme of no palpable pulse during inspiration while the formal definition only requires a decrease of systolic blood pressure by 10 mmHg during inspiration [28]. Even though first described in constrictive pericarditis, Pulsus paradoxus can also be found in pulmonary embolism as well as cardiac tamponade and other causes for increased intrathoracic pressure such as severe asthma [28]. Physiologically inspiration reduces intrathoracic pressure, increases blood pooling in the lung and consequently reduces left ventricular filling and cardiac output [23, 29]—the mechanisms that decrease systolic blood pressure during inspiration even further in events such as cardiac tamponade and pulmonary embolism are still debated [28].

#### 3.2.3 Inspection and Palpation

Subcutaneous emphysema [30] or asymmetrical breathing patterns are signs of tension pneumothorax. Regarding obstructive shock, hemoptysis is commonly seen in pulmonary embolism [31]. Classical clinical finding in aortic dissection is a pulse deficit but can only be found in a minority of cases [9]. In aortic dissection as well as Leriche’s syndrome, skin mottling or acral gangrene exposes inadequately perfused tissue [32]. In aortic dissection obvious ischemia of the lower limbs is rare [33].

#### 3.2.3 Electrocardiogram (ECG)

Common ECG-findings in pulmonary embolism include sinus tachycardia, atrial fibrillation, (in-) complete right bundle branch block, a S1Q3T3 pattern and T-wave inversion in V1-4 but are all reflective of right ventricular strain and consequently could be found in other types of obstructive shock [34]. Electrical alternans as correlate of the intrapericardial fluid collection can be found in cardiac tamponade [35]. Low-voltage as a possible ECG-correlate of tamponade occurs in pleural effusion, emphysema, obesity and anasarca [36] as well. Aortic dissection can present with signs of myocardial ischemia [9].

### 3.3 Imaging

#### 3.3.1 Sonography

**Fig. 2.**
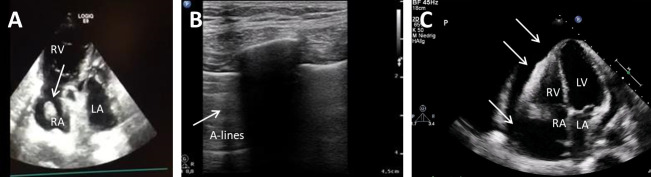
**Ultrasound in obstructive shock**. (A) TTE shows a wormously configured thrombus in the right atrium with clearly dilated right ventricles and completely emptied left ventricle. RA, right atrium; RV, right ventricle; LA, left atrium. (B) Lung sonography (LUS) shows A-lines and a missing lung sliding (white arrow), while lung on the right side is normal. (C) TTE shows a large pericardial effusion (white arrows) with swinging heart. RA, right atrium; RV, right ventricle; LA, left atrium; LV, left ventricle.

It has been shown decades ago that transthoracic echo (TTE) is sensitive and specific for cardiac tamponade and can be used to measure the effusion volume [37, 38]. Flow over the mitral- and trikuspidal-valve are dependent on in- and expiration and differences are increased in pericardial effusion [39]. Today, hemodynamic relevant tamponades are characterized by complete collapse of the right atrium (>1/3 of cardiac cycle), circumferential pericardial >2 cm (in diastole), dilated V. cava inferior (>2.5 cm, <50% inspiratory collapse), right ventricular collapse, left atrial collapse, increased mitral- and tricuspidal valve flow variations [40]. In suspected high risk pulmonary embolism which is characterized by hemodynamic instability computed tomography pulmonary angiogram (CTPA) or bedside echocardiography remain gold standard for diagnosis [41, 42]. Right ventricular failure due to pressure overload can be shown in echocardiography but cannot differentiate different types of right ventricular afterload increase. Classical echocardiographic criteria are right ventricular dilation and increased tricuspid regurgitation (velocity >2.7 ms/s) [43]. A reduced tricuspid annular plane systolic excursion (TAPSE) can occur in pulmonary embolism as well [44]. The most specific if not sensitive echocardiographic signs for pulmonary embolism are the “60/60 sign”, McConnell sign [45] and right heart thrombi [46]. On the other hand, missing signs of right ventricular overload can exclude pulmonary embolism as the cause for hemodynamic instability [47]. If performed by a skilled operator bedside sonography can be used to accurately diagnose pneumothorax by demonstrating missing lung sliding and showing a lung point [48, 49]. If the patient is stable upright chest x-ray remains a much less operator dependent alternative [50, 51]. For visual examples see Fig. 2.

We recommend using a structured approach for the ultrasound examination, which is usefull in any shock patient and might be lifesaving in case of obstructive shock. Point of care ultrasound (POCUS) allows for rapid, real-time evaluation of cardiovascular and respiratory pathology [52]. The RUSH Protocol (Rapid Ultrasound in Shock) was developed by Perera *et al*. [53], see Table 2 (Ref. [53]).

**Table 2.** **Rapid Ultrasound in Shock (RUSH) protocol: ultrasonographic findings seen with classic shock states, modified from [53]**.

RUSH Evaluation	Obstructive shock	Hypovolemic shock	Cardiogenic shock	Distributive shock
Pump	∙ Hypercontractile heart	∙ Hypercontractile heart	∙ Hypocontractile heart	∙ Hypercontractile heart (early sepsis)
	∙ Pericardial effusion	∙ Small chamber size	∙ Dilated heart	∙ Hypocontractile heart (late sepsis)
	∙ Cardiac tamponade			
	∙ RV strain			
	∙ Cardiac thrombus			
Tank	∙ Distended IVC	∙ Flat IVC	∙ Distended IVC	∙ Normal or small IVC (early sepsis)
	∙ Distended jugular veins	∙ Flat jugular veins	∙ Distended jugular veins	∙ Peritoneal fluid (sepsis source?)
	∙ Absent lung-sliding (pneumothorax)	∙ Peritoneal fluid (fluid loss)	∙ B-Lines (pulmonary edema)	∙ Pleural fluid (sepsis source)
	∙ Stratosphere- Phenomenon (pneumothorax)		∙ Pleural or peritoneal fluid (ascites)	
	∙ Distended IVC			
Pipes	∙ DVT (pulmonary embolism)	∙ (Abdominal aneurysma)	∙ Normal	∙ Normal
	∙ Aortic dissection	∙ (Aortic dissection)	∙ Possible vasoconstricted arteries	
	∙ Complete or incomplete occlusion of the aorta distal to the renal arteries (Leriche)			

Abbreviations: RV, right ventricle; IVC, inferior vena cava; DVT, deep vein thrombosis.

#### 3.3.2 Radiology

If the patient is stable enough for radiological imaging and the diagnosis could not be clarified by ultrasound, CT might help identifying the cause for shock. CT-angiography has been proven superior in sensitivity compared to TTE/TEE for diagnosis of aortic dissection [54] or pulmonary embolism. TTE shows complications such as aortic valve regurgitation [9] or hemorrhagic cardiac tamponade [55] but if it shows the typical intimal flap it can be faster than CT regarding diagnosis [56]. Aortic dissection has been grouped into Stanford A and B ever since 1970 to derive treatment necessity straight from classification, which can be derived from CT imaging—but causal therapy in shock remains limited to surgery [40, 57].

#### 3.3.3 Blood Tests

In patients presenting with any form of shock, several laboratory tests should be considered to detect the cause for shock or complications of shock. Importantly, no lab test is specific for obstructive shock and therefore should not delay diagnosis and therapy. D-dimers is not primarily recommended in shock suspected to be caused by pulmonary embolism [42] or aortic dissection [33] as CT-angiography is the faster diagnostic tool in both cases. Upcoming diagnostic tests for aortic dissection are related to vascular damage include calponin [58], plasma matrix metalloproteinase 8 [59] and tenascin-C [60] and may enter clinical routine diagnostic in the near future. There is no diagnostic laboratory test specific for cardiac tamponade [40] or pneumothorax.

Blood tests to consider might include blood gas analysis (to detect hypoxemia and acidosis), Lactate (reduced tissue perfusion [61]), glucose (hypoglycaemia), blood cell count (anemia), procalcitonin (inflammation), coagulation and platelet count (bleeding tendency), d-dimer (pulmonary embolism [62] or aortic dissection [63]), troponin, pro-brain natriuretic peptide (myocardial infarction or peulmonary embolism [64, 65, 66]), creatinine, aspartate transaminase and aspartat-aminotransferase (end-organ damage).

## 4. Diagnostic Algorithm

**Fig. 3.**
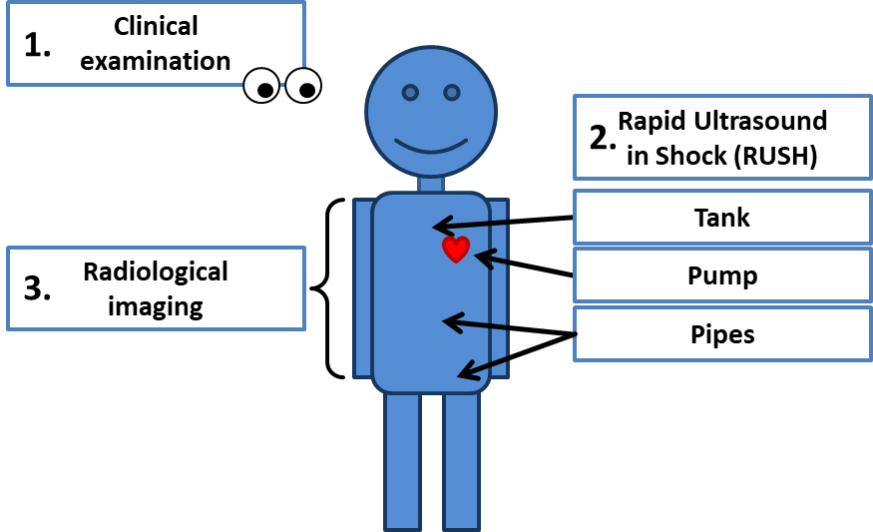
**Diagnostic algorithm in obstructive shock**.

A pragmatic approach in obstructive shock is reasonable in order to adequately identify and quickly address the causes of shock. This diagnostic algorithm includes three main steps: first clinical examination, second ultrasound studies following the RUSH protocol and third radiological imaging, see Table 3 and Fig. 3.

**Table 3.** **Diagnostic findings in obstructive shock**.

Diagnostic modality	Pericardial tamponade	Pneumothorax	Pulmonary embolism
Clinical investigation	∙ Pulsus paradoxus	∙ Silent lung on one side	∙ Signs for deep vein thrombosis
		∙ Pulsus paradoxus	∙ Venous congestion (jugular)
		∙ Venous congestion (jugular)	
		∙ Pain	
TTE	∙ Hypercontractile heart	∙ No window in cardiac echo	∙ Hypocontractile RV
	∙ Pericardial effusion	∙ Hypercontractile heart	∙ RV volume elevated
	∙ Cardiac tamponade		∙ RV strain
			∙ Cardiac thrombus
Sonography	∙ Distended IVC	∙ Absent lung-sliding	∙ Distended IVC
	∙ Distended jugular veins	∙ Stratosphere-phenomenon	∙ Distended jugular veins
Radiological imaging	∙ Not required	∙ Chest X-ray/CT confirms diagnosis	∙ CT confirms diagnosis
POCT/Lab	∙ Lactate	∙ Hypoxemia	∙ Hypoxemia
	∙ Liver failure		∙ D-dimer rule out
	∙ CVP elevated		∙ CVP elevated

Abbreviations: TTE, transthoracic echocardiogram; POCT, point of care testing; CVP, central venous pressure; IVC, inferior vena cava; CT, computed tomography; RV, right ventricle.

## 5. Treatment

### 5.1 General in Shock 

Shock is a life threatening disease and requires immediate treatment. Since the hallmark of shock is undersupply of the whole body, treatment has to support multiple organ systems to different degrees. When shock is presumed, resuscitation should be started while investigating the cause [1]. In obstructive shock, a causal therapy (e.g., paracentesis or lysis) often is the only lifesaving intervention and must not be delayed.

### 5.2 Fluid Therapy

It is important to note that fluid therapy by itself will not be able to reverse an obstructive shock. In tension pneumothorax, venous return (preload) is limited by an increase in intrapleural and secondarily intrathoracic pressure. When intrathoracic pressure exceeds intraventricular pressure, cardiac output runs dry [67, 68] and cannot be restored by fluid therapy. Especially in obstructive shock with right heart dysfunction, volume expansion can aggravate right ventricular failure [69].

Even though obstructive shock is developed in euvolemic patients with normal myocardial function, most patients are to a certain degree volume responsive [67]. Opposing the fluid resuscitation strategies in septic shock, fluid therapy in obstructive shock therefore can only buy time for a causal therapy. Choosing the right clinical surrogate for evaluating volume responsiveness however is hard and the gold standard of measuring cardiac output is dependent on the availability of advanced hemodynamic monitoring or experience in cardiac ultrasound. Passive leg raise maneuver should be used in order to test fluid responsiveness in obstructive shock. This intervention can be performed rather safely even in patients in shock [70]. Performing a passive leg raise maneuver correctly however might be challenging in unstable patients since effects on hemodynamics are transient [71]. In case passive leg raise maneuver is not feasible, a mini-fluid challenge of 100–200 mL crystalloid fluid over 10 minutes can be used in order to test volume responsiveness. Larger volumes should be avoided since fluid overload negatively impacts the outcome [72].

### 5.3 Vasopressors

Like in most critically ill patients, vasopressors are frequently used in patients with shock. While vasoplegia is a key feature in septic and anaphylactic shock, it is not in obstructive shock [73], which is more similar to cardiogenic shock presenting with centralization and elevated total vascular resistance. Here, elevated afterload can decrease cardiac output and aggravate the shock [67]. Still, maintaining a mean arterial pressure of 65 mmHg seems reasonable in shock [73] which might necessitate some vasopressor therapy. There are concerns with increased pulmonary resistance in obstructive shock like pneumothorax which might aggravate shock [67]. Some data suggests that vasopressin might not affect pulmonary resistance [74, 75] therefore offering a superior vasopressor in obstructive shock. There are however also studies suggesting a similar inert effect of norepinephrine on pulmonal vascular resistance [69] and the Guideline of the European Society of Cardiology suggests norepinephrine as first line vasopressor in obstructive shock caused by pulmonary embolism [42].

### 5.4 Inotropes

In obstructive shock, low cardiac output is not caused by myocardial dysfunction. Therefore, inotropes are often not required for stabilizing patients in obstructive shock and can be detrimental since inotropes can cause vasodilatation [76] and arrhythmias [77]. Inotropes like dobutamine or levosimendan might be however reasonable in case of chronically reduced cardiac function or in patients with increased pulmonary afterload as seen in pulmonary embolism [78, 79] or pulmonary hypertension [79, 80]. Also, inotropes in conjunction with fluid therapy might be able to increase cardiac output in obstructive shock [67].

### 5.5 Mechanical Ventilation

Patients in severe shock are frequently intubated [81]. Mechanical ventilation by itself increases afterload of the right ventricle [82], similar in nature to tension pneumothorax [67]. This fact highlights the potential detrimental effects of high pressure ventilation in obstructive shock. In case of mechanical ventilation, a low-volume (following recommendations for ARDS) and low PEEP (positive end expiratory pressure) ventilation should be aimed for in patients with shock due to high right ventricular afterload [83]. Since hypercapnia can further increase pulmonary resistance, it should be avoided [84].

### 5.6 Extracorporeal Membrane Oxygenation (ECMO)

Venoarterial extracorporeal membrane oxygenation can be a lifesaving therapy in obstructive shock caused by pericardial tamponade [85, 86]. It is important to remember that ECMO might also cause pericardial effusion [87]. There are several reports on decreased venoarterial ECMO flow in case of development of obstructive shock in neonates [88] as well as in adults [89]. On the other hand, it has been suggested that RV dysfunction can be improved by venovenous ECMO in case of pulmonary embolism by reduction on hypoxia and hypercapnia induced pulmonary vasoconstriction [90]. Similarly, venovenous ECMO unloads the right ventricle in patients with pneumonia by reducing mean pulmonary artery pressure [91]. On the other hand, venovenous ECMO is contraindicated in severe right heart failure since oxygenated blood will not be pumped into the systemic circulation [92]. Therefore, ECMO configuration and cannula placement has to be carefully evaluated in obstructive shock.

## 6. Specific

### 6.1 Pulmonary Artery Embolism

In pulmonary embolism, treatment is guided by short term (in-hospital or 30 day) mortality as proposed by the European Society of Cardiology [42]. Importantly, all patients with hemodynamic instability are considered high risk and need urgent reperfusion therapy either by medical thrombolysis (systemic or catheter guided) or surgical embolectomy [93, 94, 95, 96]. Since best reperfusion strategy depends on patient specific factors including severity of disease and bleeding risk an interdisciplinary team approach is advocated in order to optimize outcomes and might be best organized as a pulmonary embolism response team (PERT) [97]. Independent of the reperfusion strategy, the underlying disease causing PE (carcinoma, blood diseases, etc.) should be investigated and long term anticoagulation should be evaluated.

### 6.2 Pericardial Tamponade

In patients with acute pericardial tamponade, cardiac index improves and right atrial pressure decreases after pericardiocentesis [98]. The European Society of Cardiology suggests percutaneous pericardiocentesis in acute tamponade [40] can be safely performed using echocardiographic or fluoroscopic guidance. In post-cardiotomy patients (or those with recurrent effusion), surgical pericardiocentesis is frequently indicated. Caution might be reasonable in patients with very high bleeding risk or in those with severe pulmonary hypertension, since pericardiocentesis might lead to dilatation of the right ventricle with poor prognosis [99]. A recent review however found the pericardial decompression syndrome (PDS) to be a rare complication in pericardiocentesis [100].

### 6.3 Tension Pneumothorax

Unstable patients with tension pneumothorax require immediate intervention. A needle decompression is a fast and feasible treatment, provided the operator is trained and uses the right equipment [101, 102]. Chest tube thoracostomy is the treatment of choice in the hospital [103, 104]. Noteworthy, spontaneous pneumothorax rarely leads to a tension pneumothorax due to the lack of a precipitating cause (mechanical ventilation, lung disease or trauma) [22]. Further diagnostics are frequently indicated in patients with tension pneumothorax in order to identify the underlying disease.

## 7. Conclusions

Obstructive shock is a life threatening pathological condition. Since the patient typically cannot be stabilized unless the underlying cause for shock is resolved, exact diagnosis is eminent. We suggest a structured approach in three steps including clinical examination, ultrasound examination using the RUSH protocol and radiological imaging if needed.
